# The Roles of High Mobility Group Box 1 in Cerebral Ischemic Injury

**DOI:** 10.3389/fncel.2020.600280

**Published:** 2020-12-15

**Authors:** Xiaoyun Gou, Junjie Ying, Yan Yue, Xia Qiu, Peng Hu, Yi Qu, Jinhui Li, Dezhi Mu

**Affiliations:** ^1^Department of Pediatrics, West China Second University Hospital, Sichuan University, Chengdu, China; ^2^Key Laboratory of Obstetric & Gynecologic and Pediatric Diseases and Birth Defects, Ministry of Education, Sichuan University, Chengdu, China

**Keywords:** cerebral, high-mobility group box 1, ischemia, receptor for advanced glycation end products, toll-like receptor, therapeutic strategy

## Abstract

High mobility group box 1 (HMGB1) is a ubiquitous nuclear protein that plays an important role in stabilizing nucleosomes and DNA repair. HMGB1 can be passively released from necrotic neurons or actively secreted by microglia, macrophages/monocytes, and neutrophils. Cerebral ischemia is a major cause of mortality and disability worldwide, and its outcome depends on the number of neurons dying due to hypoxia in the ischemic area. HMGB1 contributes to the pathogenesis of cerebral ischemia via mediating neuroinflammatory responses to cerebral ischemic injury. Extracellular HMGB1 regulates many neuroinflammatory events by interacting with its different cell surface receptors, such as receptors for advanced glycation end products, toll-like receptor (TLR)-2, and TLR-4. Additionally, HMGB1 can be redox-modified, thus exerting specific cellular functions in the ischemic brain and has different roles in the acute and late stages of cerebral ischemic injury. However, the role of HMGB1 in cerebral ischemia is complex and remains unclear. Herein, we summarize and review the research on HMGB1 in cerebral ischemia, focusing especially on the role of HMGB1 in hypoxic ischemia in the immature brain and in white matter ischemic injury. We also outline the possible mechanisms of HMGB1 in cerebral ischemia and the main strategies to inhibit HMGB1 pertaining to its potential as a novel critical molecular target in cerebral ischemic injury.

## Introduction

High-mobility group (HMG) proteins were first isolated from the chromatin of calf thymus by Goodwin et al. (1973). HMG proteins are named based on their mobility; they migrate rapidly and show no signs of aggregation in polyacrylamide gel electrophoresis systems (Goodwin et al., 1973). HMG proteins include three superfamilies, namely HMGB, HMGA, and HMGN. The HMGB family includes the high-mobility group box 1 (HMGB1) (Bustin, 2001). The functional motif of the HGMB1 protein is the HMG-box that can recognize individual DNA structures from chromatin in a sequence-independent manner.

The HMGB1 protein localizes in the nucleus in most cells. As architectural elements of chromosomes, HMGB1 binds to DNA junctions to stabilize loop structures in chromatin. HMGB1 also bends DNA to promote protein assembly on specific DNA targets (Bianchi et al., 1992). In addition, the HMGB1 protein is an architectural transcription factor that regulates special gene transcription by interacting with chromosomal or nuclear proteins (Grosschedl et al., 1994; Zlatanova et al., 1999; Bianchi and Agresti, 2005).

Besides performing functions in the nucleus, HMGB1 is secreted into the extracellular medium by certain cells, playing a significant role in inflammation (Tang et al., 2010a,b). Cerebral ischemia is a major cause of mortality and disability worldwide, and its outcome depends on the number of neurons dying due to hypoxia in the ischemic area. HMGB1 contributes to the pathogenesis of cerebral ischemia via mediating neuroinflammatory responses to cerebral ischemic injury (Singh et al., 2016). It has been reported that HMGB1 may function as a pro-inflammatory molecule, especially through alarmin-driven inflammatory feedback mechanisms to further exacerbate the damage during cerebral ischemic injury (Singh et al., 2016). Moreover, HMGB1 acts as a damage-associated molecular pattern molecule (DAMP) in the extracellular environment, signaling to peripheral immune cells such as monocytes and macrophages, and thus initiating the local inflammatory response after cerebral ischemic injury (Singh et al., 2016).

Numerous studies have shown that HMGB1 has a detrimental role in the acute stage of cerebral ischemic injury in adults. However, the role of HMGB1 in the immature brain after hypoxic ischemia (HI) remains largely uninvestigated. Additionally, apart from the cerebral cortex, HMGB1 has a complex role in the white matter region after cerebral ischemic injury. Recent emerging studies have investigated the effect of HMGB1 on the immature brain and white matter ischemic injury (Hayakawa et al., 2013; Zhang et al., 2016; Choi et al., 2017; Frasch and Nygard, 2017; Chen et al., 2019; Hei et al., 2019; Le et al., 2019; Sun et al., 2019).

In this review, we summarize the research on HMGB1 in cerebral ischemia, especially focusing on the role of HMGB1 in HI in the immature brain and in white matter ischemic injury. Moreover, we discuss the possible mechanisms of the role of HMGB1 in cerebral ischemia and the key approaches to inhibit HMGB1 as a potential novel molecular target in cerebral ischemia.

## HMGB1 Biology

**Figure 1:**
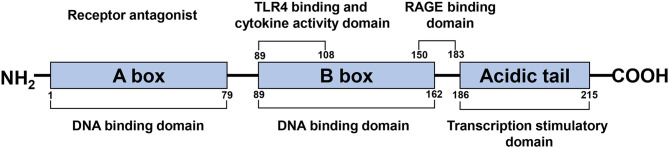
Schematic structure of the HMGB1 protein. HMGB1 is a single polypeptide chain consisting of 215 amino acids, containing two DNA binding domains (HMG boxes A, B), a N-terminal and a C-terminal. A box and B box function as RAGE receptor antagonist and cytokine, respectively.

HMGB1 is a single polypeptide chain consisting of 215 amino acids and containing two DNA binding domains (HMG boxes A and B), an N-terminal, and a C-terminal (Figure 1) (Bustin, 2001; Thomas and Travers, 2001). As a ubiquitous nuclear protein, HMGB1 acts as a non-histone chromosome binding protein. Under physiological conditions, HMGB1 in the nucleus binds to DNA non-specifically, stabilizes nucleosomes, and assists in DNA repair via identifying damaged DNA fragments and removing them (Tang et al., 2016). Cell injury, necrosis, or activation caused by certain pathological conditions such as ischemia result in the transfer of HMGB1 from the nucleus to the cytoplasm or extracellular space, due to the separation of HMGB1 from the damaged DNA (Andersson et al., 2018).

HMGB1 is thought to be involved in the pathogenesis of a variety of inflammatory diseases (e.g., sepsis) and autoimmune diseases, such as rheumatoid arthritis (Kokkola et al., 2002; Andersson and Harris, 2010; Li et al., 2010). Moreover, HMGB1 is highly expressed in certain primary tumors, including melanoma, breast cancer, and pancreatic cancer (Ellerman et al., 2007). In addition, HMGB1 is linked to some diseases characterized by cell damage and death such as diabetes and Alzheimer's disease (Nogueira-Machado et al., 2011).

**Figure 2:**
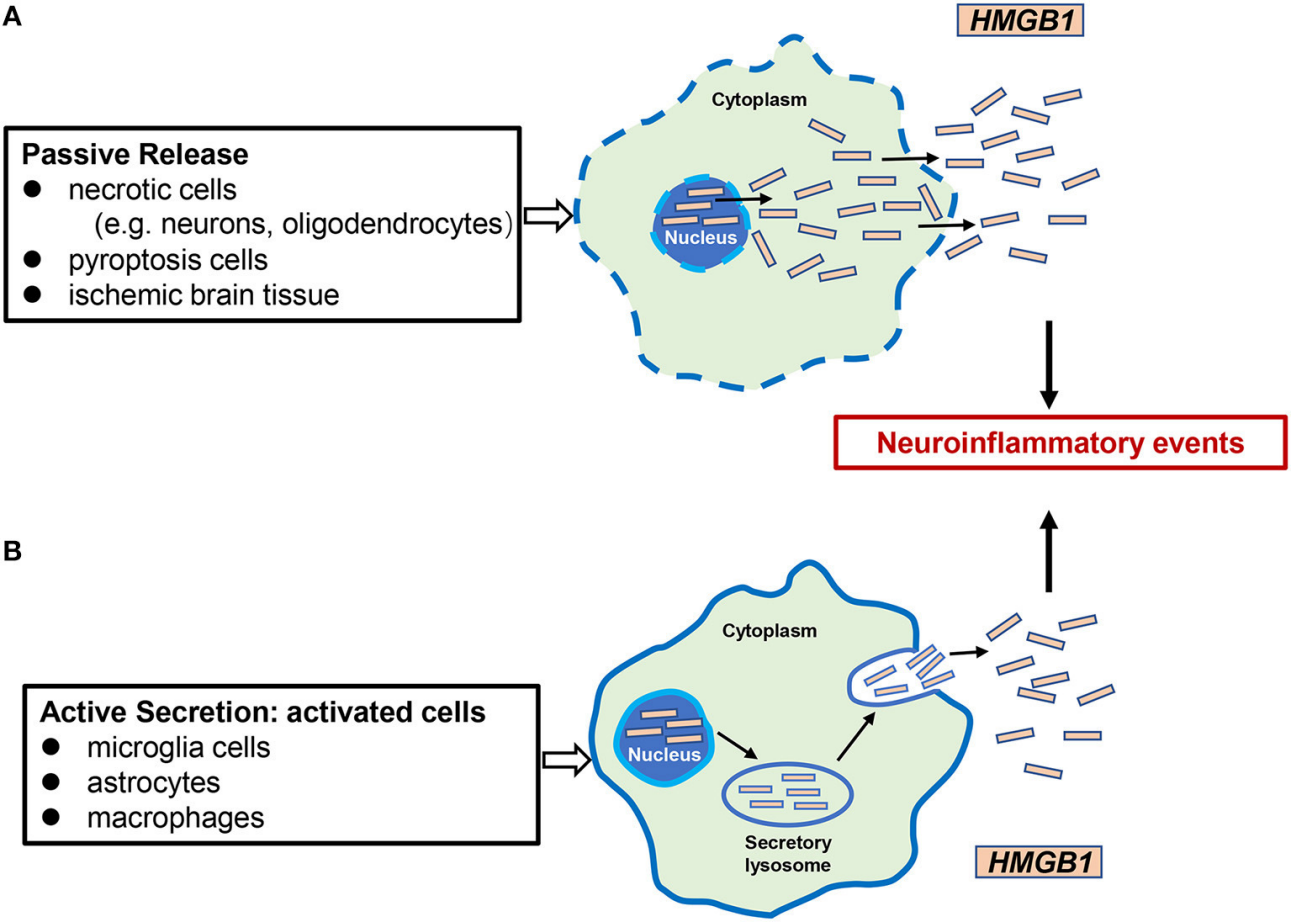
Mechanisms of HMGB1 release in cerebral ischemia. There are two mechanisms of HMGB1 from intracellular to extracellular in cerebral ischemic injury: **(A)** passive release, occurring mainly in necrotic cells (e.g., neurons, oligodendrocytes), pyrocytes, and ischemic brain tissue; **(B)** active secretion, which occurs mainly in activated microglia cells, astrocytes, and innate immune cells such as macrophages. Extracellular HMGB1 functions as a proinflammatory factor, leading to neuroinflammatory events.

HMGB1 was identified in 1999 as an important extracellular mediator in the inflammatory process (Wang et al., 1999). It can be passively released from necrotic cells such as neurons and enter the extracellular milieu by simple diffusion (Scaffidi et al., 2002). Furthermore, HMGB1 is actively secreted in the extracellular medium by certain cells such as microglia, macrophages/monocytes, and neutrophils (Gardella et al., 2002; Tang et al., 2007a; Xiong et al., 2016; Sun et al., 2019; Kim and Lee, 2020). Recent studies have shown that HMGB1 is also released through extracellular vesicles such as exosomes derived from cultured astrocytes (Ma et al., 2019). In the progression of cerebral ischemia, the passive release and active secretion of HMGB1 are not completely independent but are mutually causal. HMGB1 passively released extracellularly causes microglia and other cells to actively secrete more HMGB1, aggravates inflammation and tissue damage, and contributes to inflammatory cascades (Singh et al., 2016). After release, HMGB1 can bind to its target receptors, such as TLR-2, TLR-4, or the receptor for advanced glycation end products (RAGE), activating the expression and release of additional pro-inflammatory mediators through the positive feedback loop of NF-κB signaling pathways. These pro-inflammatory mediators include, but are not limited to TNF-α, LPS, IFN, CpG-DNA, IL-1β, and IL-8 (Figure 2) (Andersson et al., 2002; Musumeci et al., 2014).

## The Role of HMGB1 in Brain Development

In view of the fact that research in the past decades has mainly focused on the role of HMGB1 in inflammation in the extracellular space, it is worth noting that HMGB1 is also essential for the process of life and tissue regeneration.

For instance, HMGB1 plays a key role in embryonic development and cell differentiation (Muller et al., 2004). It has been reported that HMGB1 is predominantly located in the cytoplasm during brain development, promotes neurite outgrowth, and participates in the development of early neurons (Mosevitsky et al., 1989; Zhao et al., 2011; Kang et al., 2014). In particular, HMGB1 is associated with neurogenesis and neurite extension in the early phase of brain development and plays a dual role in neural development and neurodegeneration (Fang et al., 2012).

Studies on the expression pattern of HMGB1 in the development of the murine embryonic central nervous system have shown that HMGB1 is widely expressed in the early stages of brain development and is dramatically reduced at late developmental stages (Guazzi et al., 2003). In the brain of the adult mouse, HMGB1 protein is not detected in most cells, except in areas of continuing neurogenesis such as the dentate gyrus in the hippocampus, the olfactory bulb, and the granular layer of the cerebellar cortex (Guazzi et al., 2003). In addition, HMGB1 is a critical factor for forming forebrain structures in zebrafish. The HMGB1 morphants achieved by treatment with antisense morpholinos show that neural precursor cells have an insufficient ability to survive and proliferate and decreased cell population expression of the transcription factor Pax6a in the forebrain (Zhao et al., 2011). However, the effects of HMGB1 on the developing vertebrate brain are not fully understood.

Substrate surfaces coated with purified HMGB1 can promote adhesion and neurite growth during embryonic days 17–18 in rat neurons Rauvala and Pihlaskari (1987). HMGB1 promotes neurite outgrowth of granule neurons, which are enriched in the neonatal rat cerebellum (Chou et al., 2004). Furthermore, anti-HMGB1 antibodies inhibit neurite outgrowth on laminin in a reversible manner (Merenmies et al., 1991). HMGB1 is highly expressed in immature and malignant cells and exerts an essential role in neurite outgrowth and neuronal migration in the developing nervous system by binding to RAGE (Hori et al., 1995; Fages et al., 2000). It has been found that cell migration to laminin is inhibited by using antisense oligonucleotides transfected into cells to specifically decrease the HMGB1 mRNA and protein or application of affinity-purified anti-HMGB1 antibodies (Fages et al., 2000). Moreover, compared with cells of dense cultures, cells with a motile phenotype have higher HMGB1 expression (Fages et al., 2000). These findings strongly show that endogenous HMGB1 exerts a key role in the migration of immature and transformed cells. In addition, in the mouse cerebellum during development, RAGE was co-expressed with HMGB1 and sulfoglucuronyl carbohydrate (SGC) on the surfaces of granule neurons. Anti-RAGE, anti-HMGB1, and anti-SGC antibodies all inhibited neurite outgrowth and cell migration in explant and slice cultures (Chou et al., 2004). These results demonstrated that HMGB1 interaction with RAGE and SGC may play a critical role in neurite outgrowth and cell migration.

Although the downregulation or silencing of HMGB1 expression is technically achievable, it may be unstable for neonates whose nervous system is still developing, as it may deprive the host's cells from molecules responsible for nuclear housekeeping functions (Le et al., 2019). HMGB1, as an intrinsic non-histone nuclear protein, participates in many normal physiological processes. However, its suppression or deletion may have harmful consequences. HMGB1 deficiency, for instance, can cause fatal hypoglycemia in newborn mice (Calogero et al., 1999). However, it is still unclear whether HMGB1 is involved in the neuroplasticity mechanism after cerebral ischemia in adults and whether it is related to the recovery after perinatal ischemic hypoxia. Thus, more research is needed in this regard.

## HMGB1 in Cerebral Ischemic Injury

HMGB1 protein also plays an important role in the mechanism of cerebral ischemic injury. Numerous studies have shown that ischemic brain injury in adults causes the transfer of HMGB1 from the nucleus of neuron into the brain parenchyma (Kim et al., 2006; Muhammad et al., 2008; Zhang et al., 2011). However, information about the characteristics of HMGB1 after cerebral ischemia is very limited in the immature brain. Herein, we first summarize research on the role of HMGB1 on cerebral ischemic injury in adult and premature animals in recent years.

### HMGB1's Different Redox Forms

HMGB1 can be modified into reduced or oxidized forms, which have specific cellular functions in the ischemic brain. Redox modifications of HMGB1 occur at three cysteine residues at positions 23, 45, and 106, which define the biological function of HMGB1. There are three different HMGB1 redox forms. Disulfide HMGB1 has a property of cytokine induction. It has been reported that disulfide HMGB1 can activate NF-κB signaling in microglia/macrophages (Yang et al., 2015) and increase N-methyl-d-aspartate-induced hippocampal neuronal cell death *in vitro* via its interaction with TLR4 receptors (Balosso et al., 2014). Sulphonyl HMGB1 is the terminally oxidized form of HMGB1 and was shown to resolve inflammatory reactions and reduce excessive inflammatory activity (Kazama et al., 2008). Fully reduced HMGB1 has been demonstrated to induce chemotactic responses. For example, it can induce human monocytes' migration through the interaction with CXCR4 and by forming an HMGB1-CXCL12 heterodimer (Schiraldi et al., 2012).

### HMGB1 Release in Adult Stroke

Some studies have explored the alterations of HMGB1 in adult stroke. The serum HMGB1 levels were significantly elevated in patients diagnosed with a cerebral vascular accident compared with those in healthy subjects (Goldstein et al., 2006). In addition, in an animal model of MCAO, rapid accumulation of HMGB1 was detected in the serum and cerebrospinal fluid 3 h after MCAO (Kim et al., 2006).

HMGB1 can be passively released from necrotic cells such as neurons. Kim et al. (2008) investigated the subcellular localization of HMGB1 in rat brains with ischemic injury. They found that HMGB1 is widely expressed in the normal rat brain and is located in the nucleus of neuron and oligodendrocyte-like cells. HMGB1 translocated from the nucleus to the cytoplasm of the neurons 1 h after MCAO in rats and eventually disappeared from the neurons. Similar changes occur in oligodendrocyte-like cells (Kim et al., 2008). Moreover, Qiu et al. (2008) reported that HMGB1 protein was expressed in cultured neurons, glial cells, and endothelia. Early HMGB1 translocation and release occur predominantly in neurons, which was confirmed by immunofluorescence staining with anti-NeuN and anti-HMGB1 antibodies (Qiu et al., 2008).

In addition, HMGB1 is also actively secreted by microglia, macrophages, and neutrophils, and recent studies have explored the role of HMGB1 produced by these cells in cerebral ischemia. In rat and mouse models of focal cerebral ischemia, the number of CD68-positive macrophages and myeloperoxidase-positive neutrophils, which are two of the most important neuroinflammation markers, were increased in the ischemic brain (Xiong et al., 2016). Furthermore, glycyrrhizin administration reduced neuroinflammation by inhibiting the activity of macrophages and neutrophils associated with HMGB1 release from the ischemic brain after stroke (Xiong et al., 2016). Recently, it was reported that neutrophil extracellular traps (NETs) play important roles in non-infectious diseases such as ischemic stroke. In a rat permanent MCAO model, citrullinated histone H3^+^ (CitH3, a marker of NETosis) induction in neutrophils in leptomeninges was observed soon after MCAO. HMGB1, as a NETosis inducer in the ischemic brain, contributes to NETosis-mediated neuronal death and aggravates inflammation and subsequent brain tissue damage in ischemic stroke (Kim et al., 2019b; Kim and Lee, 2020).

### HMGB1 in Immature Brain After Cerebral Ischemia

Several reports have investigated the effect of HI on the expression of HMGB1 in the immature brain. In the normal fetal sheep cerebral cortex, HMGB1 is mainly located in the nucleus of neurons (Zhang et al., 2016). After ischemic treatment (30 min of carotid occlusion) of the fetal sheep brain, HMGB1 is translocated from the nucleus of cortical neurons to the cytoplasm, which may cause HMGB1 to function as a proinflammatory factor, thus damaging the developing brain after HI (Zhang et al., 2016). Additionally, under multiple repetitive, intermittent 1-min umbilical cord occlusions lasting global ischemia episodes of the ovine fetal brain, Frasch et al. found an intracellular translocation of HMGB1 in neurons, astrocytes, and microglia (Frasch and Nygard, 2017). A number of methodological differences in quantification of HMGB1 translocation may explain the contrasting findings in the dominant neuronal HMGB1 localization.

Moreover, in an animal model of neonatal hypoxic-ischemic brain injury, the authors studied the cell types involved in HMGB1 translocation and release, and found that the translocation and release of HMGB1 were observed in neurons in the ipsilateral-HI hemisphere in rats, while it was not observed in astrocytes and microglia in the same area (Chen et al., 2019). Nuclear and cytoplasmic translocation of HMGB1 occurred immediately after hypoxic-ischemic brain injury in the ipsilateral-HI hemisphere of newborn rats, and the release of HMGB1 from the cytoplasm to the extracellular space was detected as early as 6 h after HI (Chen et al., 2019). Therefore, changes in HMGB1 expression and subcellular localization after ischemic injury in neonatal brains are consistent with those in adult brains and suggest that HMGB1 could be an early sensitive indicator of neonatal hypoxic-ischemic brain injury.

In a mouse model of neonatal HI brain injury, HMGB1 was partially transferred from the nucleus of microglia to the cytoplasm after HI insult, which was involved in microglia-mediated neuroinflammation (Le et al., 2019). In this case, resveratrol exerted a neuroprotective role by inhibiting the nucleoplasmic translocation and extracellular release of HMGB1 and the activation of HMGB1/TLR4/MyD88/NF-κB signaling. These data indicated that HMGB1 participates in resveratrol's anti-neuroinflammatory effect on the immature brain (Le et al., 2019). Moreover, HMGB1 contributes to neuronal injury in a perinatal rat model of HI brain damage by modulating the polarization of M1/M2 microglia in the cerebral cortex (Sun et al., 2019). The expression of HMGB1 and the number of both M1 and M2 microglia were increased at 72 h after brain injury (Sun et al., 2019). Treatment with glycyrrhizin, an inhibitor of HMGB1, remarkably inhibited M1 microglia polarization and motivated M2 microglia polarization, meanwhile relieving brain edema and infarction size. These data indicate that HMGB1 may cause an imbalance of M1/M2 microglia polarization in the cortex and therefore result in neuronal injury after neonatal HI brain damage (Sun et al., 2019).

### HMGB1's Protective Role in Cerebral Ischemic Injury

HMGB1 has a dual role after cerebral ischemic injury. In the acute phase, it acts as a pro-inflammatory mediator, amplifying the damage of ischemic tissues by activating microglia, enhancing inflammation, and increasing the permeability of the blood-brain barrier (BBB). In the late stage of ischemic injury, it mainly participates in the restoration and reconstruction process through astrocytes in the affected brain and stimulates neurovascular repair. Recent emerging studies have assessed the possible beneficial roles of HMGB1 and implications thereof in the context of brain repair (Hayakawa et al., 2012, 2014; Chen et al., 2014).

Schlueter et al. used a spheroid model of endothelial cells *in vitro* and found that exogenous HMGB1 induces endothelial cell migration and germination in a dose-dependent manner (Schlueter et al., 2005). In a mouse model of focal cerebral ischemia, reactive astrocytes in the peri-infarct cortex upregulated HMGB1 on the fourteenth day after stroke, accompanied by the accumulation of endogenous endothelial progenitor cell (EPCs). Inhibition of HMGB1 by siRNA 5 days post-stroke *in vivo* blocked the EPC response, reduced peri-infarct angiogenesis, and aggravated neurological deficits (Hayakawa et al., 2012). These findings indicated that HMGB1 released by reactive astrocytes promotes EPC-mediated neurovascular remodeling during stroke recovery (Hayakawa et al., 2012). Moreover, inhibition of reactive astrocytes with fluorocitrate has a significant decrease in HMGB1 and obstructs neurovascular remodeling and recovery after focal cerebral ischemia in mice (Hayakawa et al., 2010). Furthermore, reactive astrocytes dramatically promoted the adherence between EPCs and cerebral endothelial cells by releasing soluble HMGB1 which then upregulated endothelial expression of RAGE via Egr1 signaling (Hayakawa et al., 2014). Besides participating in endogenous EPC-mediated neurovascular remodeling during stroke recovery, HMGB1 also involves in exogenous EPC-mediated stroke recovery. It was reported that HMGB1 upregulation promoted exogenous human peripheral blood-derived (hPB)-EPCs-mediated stroke recovery by modulating paracrine function of hPB-EPCs in transient MCAO mice (Chen et al., 2014).

HMGB1 is not only involved in damage to the cerebral cortex but also in the damage to the white matter region of the brain in cerebral ischemic injury. However, there is a paucity of research on the cellular and molecular mechanisms of ischemic white matter injury. Recent studies have investigated the pathomechanism of ischemic white matter injury.

In a diffuse ischemic bilateral common carotid artery stenosis model in mice, matrix-metalloproteinase 9 (MMP-9) produced by oligodendrocyte progenitor cells (OPCs) disrupts the BBB to aggravate ischemic injury (Seo et al., 2013). In contrast, brain-derived neurotrophic factor (BDNF) released by astrocytes boosts proliferation of oligodendrocyte lineage cells and OPC differentiation to improve ischemic white matter injury (Miyamoto et al., 2015). These studies suggest that a variety of cell types participate in the causative pathological events. Moreover, in a subcortical stroke animal model, administration of various therapeutic approaches, such as extracellular vesicles, BDNF, or adipose-derived mesenchymal stem cells (Otero-Ortega et al., 2015, 2017; Ramos-Cejudo et al., 2015), may enhance white matter restoration by mediating oligodendrocyte differentiation and myelin formation.

In addition, several studies have provided interesting ideas on identifying the role of HMGB1 in ischemic white matter injury, which remain controversial. On one hand, some studies have shown a destructive role of HMGB1 in the white matter (Hei et al., 2019); on the other hand, it has been reported that HMGB1 may be a positive factor in promoting white matter recovery (Hayakawa et al., 2013; Choi et al., 2017).

HMGB1 works as a DAMP that exacerbates acute brain injury after a stroke. It has been reported that HMGB1 and its downstream signaling TLR4/NF-κB p65 are continuously activated in the optic tract (OT) area of white matter in animal models of chronic cerebral hypoperfusion (CCH)-induced white matter lesions (Hei et al., 2019). Anti-HMGB1 neutralizing antibody can partly suppress the activation of inflammatory responses, ameliorate CCH-induced OT injuries, and facilitate visual-guided behavioral deficits (Hei et al., 2019).

However, recent studies demonstrate that HMGB1 plays an unexpectedly beneficial role during white matter restoration. Choi et al. (2017) identified HMGB1 derived from damaged oligodendrocytes (OLs), as an endogenous ligand of TLR2, which stimulates TLR2 to support the survival of neighboring OLs. Application of recombinant HMGB1 *in vitro*, in a TLR2-dependent way, ameliorated the extent of oxygen-glucose deprivation-induced OL death (Choi et al., 2017). Furthermore, in an animal model of focal white matter stroke, injection of endothelin-1 was administered with a co-injection of glycyrrhizin, a specific HMGB1 inhibitor, resulting in aggravation of demyelinating pathology *in vivo* (Choi et al., 2017). These results suggested that HMGB1 released by ischemic insult acts as an autocrine trophic factor to provide protective effects in myelinated white matter under ischemia (Choi et al., 2017). Consequently, HMGB1 and TLR2 may be novel targets for therapeutic development in ischemic white matter disease. Moreover, in a mouse model of focal demyelination in the corpus callosum, HMGB1 from reactive astrocytes was upregulated, along with the accumulation of EPCs that expressed basic fibroblast growth factor and trophic factors such as BDNF (Hayakawa et al., 2013). In particular, inhibition of HMGB1 expression with siRNA *in vivo* remarkably decreased EPC accumulation and caused proliferation of endothelial cell numbers in the damaged white matter (Hayakawa et al., 2013). These findings indicate that HMGB1 secreted by reactive astrocytes may encourage EPCs to promote damaged tissue restoration after white matter injury (Hayakawa et al., 2013).

The duality of HMGB1 in cerebral ischemia have brought on challenges linked to the therapeutic translation. A later timepoint may be suitable for designing HMGB1-directed therapies. In addition, the HMGB1-mediated angiogenesis effect may be a viable target for enhancing recovery several days after stroke onset.

## The Mechanisms of HMGB1 in Cerebral Ischemia

At present, the key role of HMGB1 in cerebral ischemia has been widely accepted, but the specific mechanisms are not yet fully understood. However, we will briefly discuss possible mechanisms next.

### Regulation of HMGB1 in Inflammation

HMGB1 is a recognized pro-inflammatory factor for ischemic stroke and is positively correlated with the severity of stroke in animal models and patients (Le et al., 2018). As an endogenous inflammatory mediator, HMGB1 is passively released by necrotic cells or actively secreted by macrophages/monocytes into the ischemic core, triggering and amplifying the inflammatory process. The released HMGB1 will also induce the activation of microglia, macrophages, and endothelial cells, thereby producing pro-inflammatory mediators such as TNF-α, IL-1β, and IL-8 (Wang et al., 2016). These pro-inflammatory mediators can not only recruit more immune cells from the circulatory system to the central nervous system, thereby aggravating the development of brain inflammation, but can also stimulate microglia and macrophages to actively secrete HMGB1, which plays the role of a late inflammatory mediator (Musumeci et al., 2014; Le et al., 2018).

### Blood-Brain Barrier Disruption by HMGB1 Release

A recent study reported that HMGB1, as an extracellular inflammatory cytokine, aggravates inflammatory damage to the BBB destruction during brain ischemia/reperfusion (Li et al., 2018). Moreover, tissue-type plasminogen activator (tPA) is a drug for ischemic stroke, but its use is limited due to the increased risk of serious neurovascular complications, such as hemorrhagic transformation (HT) and edema (Li et al., 2018). BBB leakage is an important factor in causing tPA-related HT and edema. Administration of the HMGB1-binding heptamer peptide can inhibit HMGB1 activity and dramatically improve BBB leakage by reducing the loss of the tight junction protein occludin, therefore reducing the complications resulting from tPA (Li et al., 2018).

Another factor associated with BBB penetration is the MMP. MMPs can break down a myriad of extracellular matrix proteins. Ischemic stroke can lead to the upregulation of MMPs, which in turn leads to increased BBB permeability and even its destruction. Studies have reported that the levels of HMGB1 are closely associated with the secretion of MMP-9 (Sapojnikova et al., 2014). HMGB1 can up-regulate the expression of MMP-9 through TLR4 signal (Qiu et al., 2010).

### Other Possible Mechanisms

There are other mechanisms by which the pro-inflammatory activity of HMGB1 can become harmful during cerebral ischemia, such as autophagy, oxidative stress, the adaptive immune response, T lymphocytes, and stroke-induced immunodepression (see review Ye et al., 2019b for details).

## HMGB1 Receptors in Cerebral Ischemia Injury

**Figure 3:**
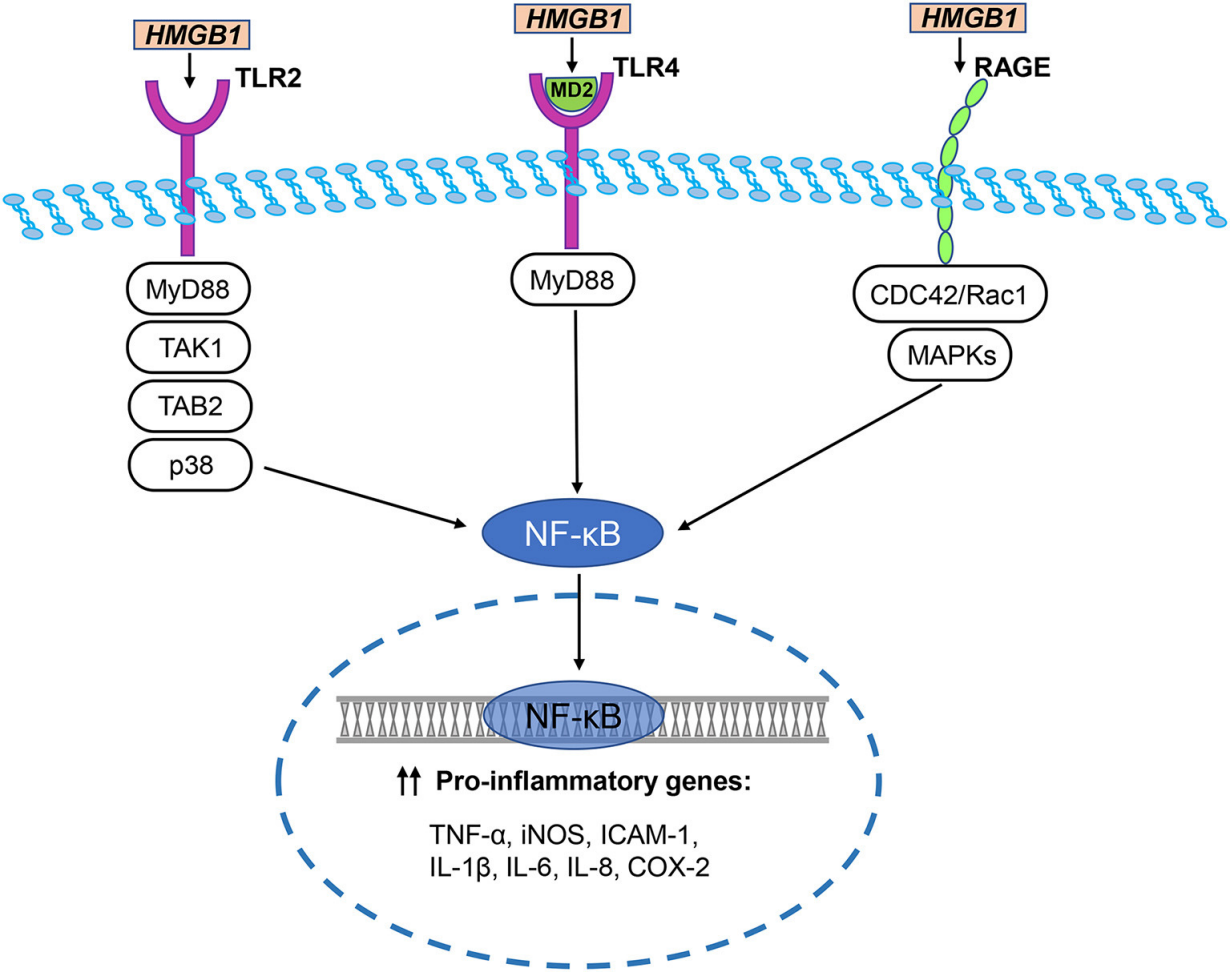
HMGB1/TLR/RAGE signaling pathways in cerebral ischemia. The primary HMGB1 receptors involved in ischemic brain injury include RAGE, toll-like receptor (TLR)-2, and TLR-4. HMGB1 directly interacts with TLR2 and stimulates increase in TLR2-mediated activation of NF-κB. Disulfide HMGB1 combines with MD-2, which forms a complex with TLR4, triggering an inflammatory response. TLR downstream signal regulators include p38, MyD88, TGF-β activated kinase 1 (TAK1), and TAK1-binding protein 2 (TAB2). They activate NF-κB to trigger an inflammatory response. Two major pathways of HMGB1-induced RAGE activation are CDC42 / Rac1 and multiple mitogen-activated protein kinases (MAPKs), which ultimately lead to NF-κB activation. NF-κB translocates to the nucleus and initiates the transcription of several pro-inflammatory genes, including TNF-α, iNOS, ICAM-1, IL-1β, IL-6, IL-8, and COX-2.

HMGB1 regulates many neuroinflammatory events by interacting with different cell surface receptors. The primary HMGB1 receptors involved in ischemic brain injury include RAGE, toll-like receptor (TLR)-2, and TLR-4. These receptors are widely expressed in neurons, astrocytes, and microglia of the central nervous system (Figure 3) (Ding and Keller, 2005a; Carty and Bowie, 2011).

### Receptor for Advanced Glycation End Products (RAGE)

RAGE is a multi-ligand signal transduction receptor for the immunoglobulin superfamily of cell surface molecules. The transmembrane and cytoplasmic domains of RAGE determine its signal transduction and induction characteristics (Bierhaus et al., 2005). RAGE is expressed in multiple cell types, such as neurons, endothelial cells, and various tumor cells (Yang et al., 2005). RAGE is expressed in neurons and glial cells in the brain (Deane et al., 2003; Arancio et al., 2004; Bierhaus et al., 2005). Interestingly, RAGE is also expressed in peripheral immune cells, such as macrophages, monocytes, and dendritic cells (Manfredi et al., 2008; Xanthis et al., 2009; Akirav et al., 2012). Studies have shown that the alarmins, which are released by the brain, activate peripheral immune cells via the RAGE signaling pathway, consequently inducing a sterile immune response (Liesz et al., 2015).

Hori et al. (1995) identified the binding of HMGB1 to RAGE in a study researching RAGE ligands. During the development of the rat brain, RAGE is highly expressed in cerebellum and hippocampal neurons and participates in neurite growth which is mediated by HMGB1 (Hori et al., 1995). RAGE and HMGB1 co-localize in the brain, and anti-RAGE antibody inhibits the expansion of cortical neuron neurite outgrowth induced by HMGB1 (Hori et al., 1995). Two major pathways of HMGB1-induced RAGE activation are the CDC42/Rac1 and multiple mitogen-activated protein kinase pathways, which ultimately lead to NF-κB activation (Huttunen et al., 1999). The typical NF-κB response gene is involved in the inflammatory response (Fiuza et al., 2003), so RAGE activation induced by HMGB1 can initiate and maintain the proinflammatory phenotype.

The pathogenic mechanisms underlying many diseases, such as neurodegenerative diseases, inflammatory diseases, diabetic complications, and cancer, involve RAGE (Rauvala and Rouhiainen, 2007). In particular, HMGB1 expression was found in the serum samples of stroke patients (Muhammad et al., 2008). Similarly, HMGB1 release was observed in ischemic brain tissue samples from mice, and the application of HMGB1 A box, an antagonist of RAGE, alleviated ischemic brain injury (Muhammad et al., 2008). This provides evidence for the functional role of HMGB1 and RAGE in a mouse model of cerebral ischemia. In addition, increased expression of RAGE was found in biopsy samples of human unilateral cerebral infarction and in the ischemic cerebral hemisphere of MCAO rats (Zhai et al., 2008). Upregulated expression of RAGE can be detected in vascular cells 12 h after cerebral ischemia; however, upregulation of RAGE expression occurs 3 to 7 days after ischemic injury in hippocampal neurons and glial cells (Kamide et al., 2012). Qiu et al. (2008) used a mouse MCAO model to detect the presence of HMGB1 receptor RAGE in brain tissues and in cultured primary neurons and astrocytes *in vitro* and found that RAGE expression levels were significantly increased in the ischemic preinfarction area of damaged brain cortex compared with the normal brain. These results further support the critical role that RAGE plays in the HMGB1 signal transduction pathway, which contributes to HMGB1's key role in pro-inflammation in ischemic brain injury.

A previous study further assessed the role of RAGE in cerebral ischemic injury. In this study, blood endogenous secretory RAGE (esRAGE), a truncated variant of RAGE isoforms, remarkably inhibited transient brain ischemia-induced neuronal cell damage and apoptosis in the mouse bilateral common carotid artery occlusion model (Shimizu et al., 2020). The neuroprotective effects of esRAGE in ischemia are associated with its transfer into the brain through the BBB by RAGE on endothelial cells (Shimizu et al., 2020). These findings indicate that endothelial RAGE has two distinct roles in ischemia, namely, as an inducer of vascular and neuronal injury and as a transporter of esRAGE, a neuroprotector, to the brain (Shimizu et al., 2020).

In addition, HMGB1 release and elevated RAGE expression plays a significant role in the pathogenesis of hypertension, hyperlipidemia, and diabetes mellitus, which are the most common risk factors for atherosclerosis and contribute to cerebral vessel occlusion during ischemic stroke (Richard et al., 2017). For instance, angiotensin receptor blockers, such as telmisartan, irbesartan, and candesartan, can regulate the HMGB1-RAGE axis and inhibit RAGE expression, which exert a beneficial effect on prevention and treatment of ischemic stroke (Kikuchi et al., 2013).

### Toll-Like Receptors (TLRs)

TLRs are a class of transmembrane proteins that have multiple members in humans and mice. They are structurally composed of extracellular domains, transmembrane domains, and cytoplasmic domains. The extracellular domain participates in ligand recognition, and the cytoplasmic domain binds to the adapter protein MyD88 to participate in signal transduction (Chang, 2010). TLR-mediated signals can stimulate cell proliferation and pro-inflammatory cytokine secretion (Brasier, 2006).

HMGB1 has different redox forms and can bind to specific TLR receptors (Caso et al., 2007; Tu et al., 2010). For example, disulfide HMGB1 combines with MD-2, which forms a complex with TLR4, triggering an inflammatory response (Yang et al., 2015). TLR2 and TLR4 are considered to be key HMGB1 receptors that induce inflammation of macrophages and lymphocytes. HMGB1 directly interacts with TLR2 and stimulates increase in TLR2-mediated activation of NF-κB (Park et al., 2006). In multiple cell lines, anti-TLR2 antibodies suppress the secretion of TNF-α and IL-8 induced by HMGB1 (Yu et al., 2006). Moreover, the brain damage and neuronal defects of TLR2- and TLR4-knockout mice were reduced after cerebral ischemia caused by MCAO (Caso et al., 2007; Tang et al., 2007b). Further, a study explored the effects of TLR2, TLR4, and RAGE in response to HMGB1 *in vivo* (van Zoelen et al., 2009). In the peritoneal lavage fluid of wild-type mice, the levels of TNF-α and IL-6 increased over time which was induced by HMGB1 (van Zoelen et al., 2009). Compared with wild-type mice, 2 h after intraperitoneal injection of HMGB1, the concentrations of TNF-α and IL-6 were decreased in the peritoneal lavage fluid of the TLR4^−/−^ and RAGE^−/−^ mice, whereas concentrations were increased in the TLR2^−/−^ mice (van Zoelen et al., 2009). These data indicate that HMGB1 induces inflammatory factor release through TLR4 and RAGE-dependent mechanisms *in vivo*, and that TLR2 and TLR4 play different roles in the HMGB1 signaling pathway. TLR downstream signal regulators include p38, MyD88, TGF-β-activated kinase 1 (TAK1), and TAK1-binding protein 2, which are thought to be involved in cerebral ischemic injury induced by HMGB1 (Downes et al., 2013). In the experimental filament-induced MCAO mouse model, compared with wild-type mice, MyD88 knockout mice have smaller stroke lesions (Downes et al., 2013). In contrast, another study found that in the MCAO model, the interruption of MyD88 signaling did not reduce the infarct size, suggesting that there may be additional or alternative downstream adapters in TLR signaling during cerebral ischemia (Famakin et al., 2011).

In addition, the NLRP3 inflammasome is associated with HMGB1 activation during cerebral ischemia. For example, in a mouse model of focal cerebral ischemia-reperfusion injury, NLRP3 inflammasome inactivation was mediated by inhibition of the TLR4/NF-κB signaling pathway, which resulted in HMGB1 downregulation, consequently alleviating brain damage in ischemic stroke (Ye et al., 2019a). Moreover, in a mouse model of global cerebral ischemia, the NLRP3/ASC/caspase-1 signaling pathway was involved in ischemic brain injury (Li et al., 2020). Inhibition of the NLRP3 inflammasome activation may have a neuroprotective role after cerebral ischemia (Li et al., 2020). Recent research by Pan et al. (2020) revealed connections between HMGB1 and autophagy in an MCAO model in juvenile rats. HMGB1 was shown to bind to Beclin1, which is involved in the recruitment of autophagy-related proteins, to maintain autophagy. In a rat model of childhood stroke, the authors showed that treadmill exercise alleviates neurological damage by inhibiting autophagy via blocking the binding of HMGB1 to Belin1 (Pan et al., 2020). Collectively, these results indicated that HMGB1 plays an important role in regulating autophagic pathways during cerebral ischemia-reperfusion (Pan et al., 2020).

In summary, HMGB1 send signals through RAGE, TLR2, and TLR4, which are pattern recognition receptors, leading to increased inflammation after cerebral ischemic injury.

## HMGB1 as the Target of Treatment in Cerebral Ischemia

HMGB1 exhibits proinflammatory cytokine activity in the extracellular space during cerebral ischemia. As mentioned above, HMGB1 plays different roles in the early and late phases of cerebral ischemia; thus, the timing of drug administration with respect to the phase of cerebral ischemia should be considered. HMGB1 exerts a harmful effect in the early stage of cerebral ischemia and aggravates inflammation and brain tissue damage. Therefore, the early stage of cerebral ischemia is the appropriate time window for interventions against HMGB1. Based on the diverse roles of HMGB1 in a variety of pathologies, different methods have been found to inhibit the expression, release, and activity of HMGB1 to ameliorate brain damage. The main strategies to inhibit HMGB1 include: (1) blocking HMGB1 release from cells, (2) binding to HMGB1 and neutralizing its activity, (3) blocking HMGB1 activation or interaction with specific receptors, and (4) suppressing downstream signaling initiated by HMGB1 (Tsung et al., 2014).

### Small-Molecule Inhibitor of HMGB1

In recent years, glycyrrhizin, a small molecule derived from the roots and rhizomes of glycyrrhiza glabra, has been investigated for its capacity to inhibit the pathological activity of HMGB1. Glycyrrhizin binds directly to the HMG A-box and HMG B-box of HMGB1 and suppresses its chemoattractant and mitogenic activities (Mollica et al., 2007). Many experimental animal studies have suggested that glycyrrhizin can inhibit the cytokine activity of extracellular HMGB1 and prevent the occurrence of ischemia-induced cerebral injury. Indeed, it has been demonstrated that glycyrrhizin reduced infarctions and neuroinflammation in rats after cerebral ischemic injury caused by MCAO by inhibiting T-cell activity via inhibition of HMGB1 release after stroke (Xiong et al., 2016). In another study, it was found that glycyrrhizin attenuated ischemic rat spinal cord injury by reducing the levels of IL-1β, IL-6, and TNF-α, and inhibiting HMGB1 release (Gong et al., 2012). In particular, it has been found that glycyrrhizin blocked HMGB1 secretion by suppressing its phosphorylation, and hence exerted anti-inflammatory effects in the post-ischemic rat brain after MCAO (Kim et al., 2012). A recent study showed that glycyrrhizin decreases HMGB1 expression, inhibits the release of inflammation cytokines such as IL-1β, IL-6, and TNF-α initiated by HMGB1, and reduces the expression of HMGB1/TLR/NF-κB pathway-related proteins in the rat brain after cerebral ischemia/reperfusion injury (Yan et al., 2019). In addition, peroxynitrite, an important cytotoxic factor derived from superoxide and nitric oxide, activated HMGB1/TLR2/MMP-9 signaling in an ischemic-stroke rat model with delayed t-PA treatment (Chen et al., 2020). Glycyrrhizin administration inhibited peroxynitrite production, downregulated the expression of HMGB1, TLR2, and MMP-9, and thus improved neurological outcomes via relieving brain edema, BBB disruption, HT, and neuronal apoptosis. These results demonstrated that glycyrrhizin could be have an neuroprotective effect by inhibiting the peroxynitrite/HMGB1/TLR2 signaling cascades (Chen et al., 2020).

### Anti-HMGB1 Antibodies

Numerous studies have shown that HMGB1 has a detrimental role in the acute stage of cerebral ischemic injury. Early reperfusion recovery is an important step to prevents decreased cerebral perfusion in patients with cerebral ischemia. However, ischemic stimulation may activate macrophages to release the inflammatory mediator HMGB1, while reperfusion may stimulate excessive HMGB1 release into the extracellular environment, aggravating brain tissue damage, and thereafter aggravating the inflammatory response (Ye et al., 2019b).

Anti-HMGB1 treatment for ischemic brain injury has been reported to reduce inflammation and cell death in the early stages of cerebral ischemia. For instance, the inflammatory molecules TNF-α, ICAM-1, and inducible nitric oxide synthase (iNOS) are known to play a vital role in neuroinflammation. Expression of these inflammatory molecules was upregulated after recombinant HMGB1 treatment in neurons, astrocytes, and endothelial cells *in vitro* (Qiu et al., 2008). HMGB1 released from injured neurons in culture induced TNF-α mRNA expression in astrocytes, which can be dramatically inhibited by the anti-HMGB1 blocking antibody (Qiu et al., 2008).

The role of the anti-HMGB1 antibody was further confirmed *in vivo*. Treatment with an intravenous injection of neutralizing anti-HMGB1 monoclonal antibodies (mAb) significantly reduced the infarct volumes in the cerebral cortex and striatum caused by middle cerebral artery occlusion (MCAO) in rats (Liu et al., 2007). Furthermore, the administration of anti-HMGB1 mAb also ameliorated the accompanying neurological deficits, inhibited microglial activation and MMP-9 activity, and suppressed the expression of TNF-α and iNOS. These findings indicate that HMGB1 plays a critical role in the post-ischemic brain by amplifying multiple inflammatory responses (Liu et al., 2007).

In addition, Zhang et al. (2011) applied anti-HMGB1 mAb to animal models of MCAO and found that it can significantly inhibit the translocation and release of HMGB1 in cerebral cortex neurons, reduce BBB permeability, and facilitate the clearance of circulating HMGB1 from the bloodstream. Thereby, anti-HMGB1 mAb reduces the degree of BBB disruption and subsequent cerebral edema.

Similarly, the administration of a neutralizing anti-HMGB1 antibody via an intraperitoneal injection 15 min before MCAO in a mouse stroke model remarkably reduced the infarct volume and decreased the number of apoptotic cells in the periphery of the ischemic area (Muhammad et al., 2008).

These results suggest that the anti-HMGB1 antibody has a protective effect against cerebral ischemic injury.

### HMGB1 A Box

HMGB1 has two DNA-binding domains; the A box, the N-terminal part of HMGB1, is a competitive antagonist of RAGE (Bianchi and Manfredi, 2007), the B box of HMGB1 is essential for the functionality of proinflammatory cytokines (Li et al., 2003). A box has a 40% sequence homology to the B box but has no intrinsic proinflammatory activity (Yang et al., 2004). In contrast, the A box specifically competes for HMGB1 proinflammatory activity through binding sites on the surface of activated macrophages, attenuating proinflammatory cytokines release induced by HMGB1 (Yang et al., 2004; Gong et al., 2009). Therefore, the A box has an anti-inflammatory effect with therapeutic potential (Li et al., 2003). For instance, intraperitoneal injection of HMGB1 A box in mice 15 min before MCAO dramatically decreased the infarct volume (Muhammad et al., 2008).

### Soluble RAGE

RAGE has two main isoforms, one is the full-length RAGE and the other is the soluble RAGE lacking the transmembranous domain and intracellular tail, which is essential for RAGE-mediated signaling (Ding and Keller, 2005b). Soluble RAGE functions as a decoy to antagonize with full-length RAGE for ligand binding (Schmidt et al., 1994; Sárkány et al., 2011). RAGE binds to endogenous ligands, especially DAMPs, such as HMGB1 (Musumeci et al., 2014). RAGE activated by HMGB1 mediates brain macrophage activation after cerebral ischemia (Muhammad et al., 2008). Treatment with soluble RAGE intraperitoneally in a mouse MCAO model remarkably reduced the infarct volume and diminished the number of CD11b^+^ cells, a microglia/macrophage marker, which was used to evaluate soluble RAGE effect on inflammatory responses (Muhammad et al., 2008). Consequently, HMGB1-RAGE signaling may be an anti-inflammatory therapeutic target in cerebral ischemic injury.

### RNA Interference

RNA interference (RNAi) is a powerful tool for gene silencing in a variety of organisms, and it is widely used in gene function research and various therapeutic effect studies (McManus and Sharp, 2002; Dorsett and Tuschl, 2004). RNAi can be performed by plasmids encoding short-hairpin RNAs (shRNAs) or by delivering small interfering RNAs (siRNAs).

HMGB1 gene silencing, mediated by RNAi in the central nervous system, has been carried out in by several studies. For example, HMGB1 mRNA was knocked down by using a plasmid expressing the shRNA of the HMGB1 gene. HMGB1 suppression mediated by shRNA had a neuroprotective effect in the post-ischemic brain, including remarkably reduced infarct volumes (Kim et al., 2006). In particular, HMGB1 downregulation by shRNA reduced the number of activated or phagocytic microglia and repressed the release of the proinflammatory molecules IL-1β, COX-2, TNF-α, and iNOS, indicating that downregulation of HMGB1 expression has an anti-inflammatory effect in the post-ischemic brain (Kim et al., 2006). In addition, a new biodegradable non-viral gene vector, arginine-PAMAM esters (e-PAM-R), which forms a complex with siRNA, can improve the transfection efficiency of siRNA and downregulate the mRNA and protein levels of the target gene, HMGB1. In an animal model of focal cerebral ischemia, HMGB1 gene knockdown mediated by siRNA/e-PAM-R complex notably reduced infarct formation, alongside having a neuroprotective effect (Kim et al., 2010).

Since the BBB can effectively prevent the passage of macromolecular and hydrophilic drugs, the drug delivery efficiency to the central nervous system is limited. Therefore, in the animal model of MCAO, Kim I.D. et al. (2012) further optimized the administration route and adopted the non-invasive intranasal delivery for direct drug administration and delivery to the central nervous system. It was found that intranasal delivery of HMGB1-siRNA can significantly reduce the levels of HMGB1 mRNA and protein in the striatum and superior prefrontal cortex, reduce the formation of the infarct, and improve neurological and behavioral deficits (Kim I.D. et al., 2012). In another study, HMGB1-siRNA was loaded into rabies virus glycoprotein (RVG) peptide-decorated exosomes (RVG-Exo) by electroporation and then delivered into the ischemic brain by intravenous injection in an MCAO model. The authors found a more efficient decrease in HMGB1 levels, TNF-α levels, apoptosis in the brain, and infarct volume in the RVG-Exo/HMGB1-siRNA group compared to that in the unmodified exosomes/HMGB1-siRNA group, indicating that RVG-Exo with HMGB1-siRNA may represent a novel therapeutic system for ischemic stroke treatment (Kim et al., 2019a). These findings further provide evidence of a new mechanism of dynamic crosstalk between HMGB1 and cerebral ischemia.

### Others

In addition to the above strategies, several other strategies have been proposed to inhibit HMGB1, including peptides and proteins, physical methods, chemicals, and clinical drugs. For example, in the MCAO juvenile rat model, treadmill exercise inhibited cell autophagy in the ischemic penumbra by inhibiting HMGB1 translocation and its binding with Beclin1 to improve neurological function (Pan et al., 2020).

Meisoindigo, a second-generation derivative of indirubin, exerted a neuroprotective role in a mouse model of MCAO stroke by inactivating the NLRP3 inflammasome and regulating the polarization of microglia/macrophages via inhibition of the TLR4/NF-κB signaling pathway, resulting in HMGB1 downregulation (Ye et al., 2019a).

It was reported that berberine can prevent HMGB1 nuclear-to-cytosolic translocation after ischemic stroke. Metastasis-associated lung adenocarcinoma transcript 1 (Malat1) belongs to long non-coding RNAs and acts as a competitive endogenous RNA for miR-181c-5p, targeting the 3′-UTR of HMGB1 to aggravate neuroinflammation after ischemic stroke. Berberine exerts its anti-inflammatory effects through Malat1/miR-181c-5p/HMGB1 axis in stroke (Cao et al., 2020).

In addition, notoginseng leaf triterpenes (PNGL) were found to be neuroprotective in an MCAO reperfusion rat model (Xie et al., 2019). PNGL significantly reduced infarct volume, alleviated brain edema, improved neurological functions, reduced BBB damage, inhibited neuronal apoptosis and loss, and reduced the serum concentrations of the inflammatory factors IL-1β, IL-6, and TNF-α. Further studies have found that the protective effect of PNGL is mainly associated with the inhibition of inflammation triggered by HMGB1 and the inactivation of its downstream pathway, NF-κB (Xie et al., 2019).

The details of other strategies are presented in Table 1 (Hayakawa et al., 2010; Wang et al., 2010a,b, 2011; Jin et al., 2011; Li et al., 2012; Shin et al., 2014).

**Table 1:** HMGB1-targeting therapeutic strategies.

**Therapeutic strategies**	**Inhibition of HMGB1**	**Animal type**	**Occlusion type**	**Treatment timing**	**Main outcome**	**References**
**Antibody**						
Anti-HMGB1 monoclonal antibody	Release	Mouse (male, C57BL/6) Rat (male, Wistar) Rat (male, Wistar)	1. Permanent middle cerebral artery occlusion (MCAO) 2. 2 h transient MCAO 3. 2 h transient MCAO	a)15 min before MCAO b) immediately and 6 h after reperfusion c) immediately and at 6 h after reperfusion	1. Significantly reduced the infarct volume 2. Ameliorated brain infarction, significantly improved neurological deficits in locomotor function, inhibited the increased permeability of the blood-brain barrier, the activation of microglia, the expression of TNF-α and iNOS, and suppressed the activity of MMP-9 3. Reduced the blood-brain barrier permeability and inhibited the development of brain edema	1. Muhammad et al., 2008 2. Liu et al., 2007 3. Zhang et al., 2011
**HMGB1-receptor and signaling pathway inhibition**						
Soluble RAGE	Inhibition of HMGB1-RAGE pathway	Mouse (male, C57BL/6)	Permanent MCAO	15 min before MCAO plus 90 min after MCAO	Significantly reduced the infarct size and the inflammatory response	Muhammad et al., 2008
**Peptide and protein**						
A box	Activity	Rat (male, Sprague-Dawley)	1h transient MCAO	at 1 h, 3 h, or 6 h after MCAO	Reduced infarct volumes, along with remarkable improvement of neurological deficits, suppressed proinflammatory cytokine inductions	Jin et al., 2011
Fetuin-A	Release	Mouse (male, C57BL/6) Rat (male, Lewis)	Permanent MCAO	at 15, 30, or 60 min after MCAO	Reduced brain infarct volume at 24 h after MCAO, effectively attenuated (i) ischemia-induced HMGB1 depletion from the ischemic core; (ii) activation of centrally (e.g., microglia) and peripherally derived immune cells (e.g., macrophage/monocytes); and (iii) TNF production in ischemic brain tissue	Wang et al., 2010aa
**RNAi**						
HMGB1 siRNA	Expression	Rat (male, Sprague-Dawley)	1 h transient MCAO	24, 12, or 3 h prior to or 1, 3, or 6 h post-MCAO	Markedly suppressed infarct volume; recoveries from neurological and behavioral deficits	Kim I.D. et al., 2012
HMGB1 shRNA	Expression	Rat (male, Sprague-Dawley)	1 h transient MCAO	24 h before the 1 h of MCAO	Suppressed infarct size, microglia activation, and proinflammatory marker induction	Kim et al., 2006
**Chemicals**						
Ethyl pyruvate (EP)	Release and expression	Rat (male, Sprague-Dawley)	1 h transient MCAO	treatment with EP 30 min post-MCAO; delayed EP treatment from 4 days post-MCAO	Treatment with EP 30 min post-MCAO reduced ischemic infarct volume; delayed EP treatment from 4 days post-MCAO reduced HMGB1 accumulation in CSF at 7 day post-MCAO in the absence of accompanying amelioration of ischemic brain damage	Shin et al., 2014
Glycyrrhizin (Gly)	Activity and release	Rat (Sprague-Dawley) Rat (T cell-deficient nude) Mouse (C57BL/6J) Mouse (SCID)	100 min of transient MCAO in rats; 60 min of MCAO in mice	immediately before MCAO and 2 h after reperfusion; immediately post-reperfusion and 3.5 h after reperfusion; immediately post-reperfusion.	Reduced infarctions and neuroinflammation in wild-type animals; did not offer protection in nude rats and severe combined immunodeficient (SCID) mice who had no T cells, while Gly reduced infarction in both nude rats and SCID mice whose T cells were reconstituted; attenuated gene expression of IFNγ, but not IL-4 and IL-10 in CD4 T cells.	Xiong et al., 2016
**Physical method**						
Molecular hydrogen	Release	Rat (male, Sprague-Dawley)	Permanent MCAO	at 5 min after MCAO followed by hydrogen-rich saline injections at 6 h, 12 h, and 24 h	Significantly reduced infarct volume and improved neurobehavioral outcomes; dose-dependently increased the activities of endogenous antioxidant enzymes (SOD and CAT) as well as decreased the levels of oxidative products (8-iso-PGF2a and MDA) and inflammatory cytokines (TNF-a and HMGB1) in injured ipsilateral brain tissues	Li et al., 2012
**Clinical drugs**						
Atorvastatin	Expression	Rat (male, Sprague-Dawley)	Permanent MCAO	Immediately after MCAO	Dramatically improved neurological deficits, reduced brain water contents and infarct sizes at 24 h after stroke and attenuated the over-expression of HMGB1, RAGE, TLR4, and NF-κB induced by ischemia	Wang et al., 2010a
Fluorocitrate	Release	Mouse (male, ddY)	4h transient MCAO	5 days after stroke onset	A significant decrease in HMGB1-positive reactive astrocytes and neurovascular remodeling, as well as a corresponding worsening of behavioral recovery	Hayakawa et al., 2010
Parecoxib	Release	Rat (male, Sprague-Dawley)	Permanent MCAO	15 min before ischemia and again 12 h after inchemia	Significantly improved neurological deficit scores, reduced infarct volume, and decreased HMGB1 and TNF-α levels	Wang et al., 2011

## Conclusions

HMGB1 has been previously studied in the context of a variety of human diseases, such as infectious diseases, neurodegenerative diseases, and cancer. Accumulating scientific evidence suggests that HMGB1 exerts an important role in central nervous system diseases, particularly in cerebral ischemia. Several recent studies highlight the important role of HMGB1 in cerebral ischemic injury and indicated that HMGB1 participates in neuropathology and neuroinflammation. Based on previous preclinical and clinical studies, HMGB1 may be thought of as a hopeful non-invasive biomarker of cerebral ischemia. Targeting HMGB1 may represent an effective therapeutic strategy for cerebral ischemic injury. However, it is very difficult to translate the therapeutic effect obtained from experimental evidence into clinical practice. For example, under inflammatory conditions such as ischemic brain injury, the antibodies produced against HMGB1 do not cross-react with HMGB2. Thus, once HMGB1 is cleared by antibodies, HMGB2 may replace HMGB1 as a trigger for inflammation. Moreover, different redox forms of HMGB1 in patients with ischemic stroke have unclear biological functions in the perpetuation and regression of inflammation after acute ischemic brain injury. It will be a challenge to develop therapeutic strategies to regulate the redox forms of inflammatory HMGB1 that have more neuroprotective or regenerative functions. In conclusion, targeting HMGB1 may emerge as a novel therapeutic strategy for cerebral ischemic injury.

## Author Contributions

DM, JL, and XG conceptualized and designed the literature review. XG and JY drafted the initial manuscript, reviewed, and revised the manuscript. YY, XQ, and PH provided critical revisions and contributed to the final manuscript. YQ critically reviewed the manuscript for important intellectual content. All authors read and approved the final manuscript as submitted and agree to be accountable for the content of the work.

## Conflict of Interest

The authors declare that the research was conducted in the absence of any commercial or financial relationships that could be construed as a potential conflict of interest.

## Abbreviations

BBB: blood-brain barrier
BDNF: brain-derived neurotrophic factor
CCH: chronic cerebral hypoperfusion
CPF: sub-toxic chlorpyrifos
DAMP: damage-associated molecular pattern molecule
e-PAM-R: arginine-PAMAM esters
EPCs: endothelial progenitor cells
esRAGE: endogenous secretory RAGE
HI: hypoxic ischemia
HMG: high-mobility group
HMGB1: high mobility group box 1
HT: hemorrhagic transformation
iNOS: inducible nitric oxide synthase
mAb: monoclonal antibodies
Malat1: metastasis-associated lung adenocarcinoma transcript 1
MCAO: middle cerebral artery occlusion
MMP: matrix-metalloproteinase
NETs: neutrophil extracellular traps
OLs: oligodendrocytes
OPC: oligodendrocyte progenitor cell
OT: optic tract
PNGL: notoginseng leaf triterpenes
RAGE: receptors for advanced glycation end products
RNAi: RNA interference
RVG: rabies virus glycoprotein
RVG-Exo: peptide-decorated exosomes
SGC: sulfoglucuronyl carbohydrate
shRNAs: short-hairpin RNAs
siRNAs: small interfering RNAs
TAK1: TGF-β-activated kinase 1
TLR: toll-like receptor
tPA: tissue-type plasminogen activator.
